# To give the invisible child priority: Children as next of kin in general practice

**DOI:** 10.3109/02813432.2014.874133

**Published:** 2014-03

**Authors:** Frøydis Gullbrå, Tone Smith-Sivertsen, Guri Rortveit, Norman Anderssen, Marit Hafting

**Affiliations:** ^1^Research Unit for General Practice in Bergen, Uni Health, Bergen, Norway; ^2^Department of Global Public Health and Primary Care, University of Bergen, Norway; ^3^Department of Psychosocial Science, University of Bergen, Norway; ^4^Regional Research Centre for Child and Adolescent Mental Health West, Uni Health, Bergen, Norway

**Keywords:** Child of impaired parent, children as next of kin, disease prevention, family health, focus group, general practice, general practitioner, health promotion, Norway, qualitative research

## Abstract

*Objective*. To explore general practitioners’ (GPs’) experiences in helping children as next of kin of drug-addicted, mentally ill, or severely somatic ill adults. These children are at risk of long-term mental and somatic health problems. *Design*. Qualitative focus-group study. *Setting*. Focus-group interviews were conducted in western Norway with a total of 27 GPs. Participants were encouraged to share stories from clinical encounters with parents who had one of the above-mentioned problems and to discuss the GP's role in relation to helping the patients’ children. *Results*. The GPs brought up many examples of how they could aid children as next of kin, including identifying children at risk, counselling the parents, and taking part in collaboration with other healthcare professionals and social workers. They also experienced some barriers in fulfilling their potential. There were time constraints, the GPs had their main focus on the patient present in a consultation, and the child was often outside the attention of the doctors, or the GPs could be afraid of hurting or losing their vulnerable patients, thus avoiding bringing up the patients’ children as a subject for discussion. *Conclusions*. Norwegian GPs are in a good position to help children as next of kin and doctors make a great effort to support many of them. Still, support of these children by GPs often seems to depend not on careful consideration of what is best for the patient and the child in the long run, but more on short-term convenience reasons.

Children whose parents are suffering from mental health illness, substance abuse, or severe somatic disease are at risk of developing poor health and psychosocial problems. Meeting their special needs is important for health promotion and disease prevention.

- GPs are in a good position to identify children as next of kin, support parents in their parenting role, and take part in the multidisciplinary network.
- Lack of time and capacity problems are barriers for GPs in fulfilling this potential.
- GPs’ fear of jeopardizing the relationship with their patients represents an important barrier to introducing the children's situation in consultations with the parents.

## Introduction

Children whose parents are suffering from mental illness, substance abuse, or severe somatic disease are at risk of developing psychosocial problems, psychiatric diseases, and somatic diseases [1–3]. Several reports have addressed these children's special needs [4–8]. In order to secure their rights, new law paragraphs were launched in Norway on 1 January 2010 [9]. According to these laws, healthcare personnel treating patients in one of the aforementioned three groups should enquire whether they have children younger than 18 years of age, and make the necessary effort to ensure that the children receive adequate information and follow-up [9,10]. It is challenging for healthcare professionals to include the children while treating the parents [11].

According to a report from 2011, 115 000 children in Norway, or 10%, live with one or both parents with severe mental illnesses and 30 000 or 3% with one or both parents with severe substance abuse [7]. Some 15 000 children or 1% have a parent or sibling with cancer, or live with grief after the death of close family members [6]. Norway has a registered list system for general practice, and almost all inhabitants are listed [12]. On a standard general practitioner (GP) list of 1200 persons, there are approximately 39 children with these kinds of burdens.

According to McWhinney, to succeed in building a relationship with the parents in order to meet the individual's and his/her child's needs, communication ought to be patient-centred [13]. The patient-centred approach has been shown in several studies to enhance communication between patient and doctor [14,15], and the method is taught to medical students in all Norwegian medical schools.

GPs are potentially in a unique position to identify at-risk children and to ensure that they are adequately followed up, either in their own practice or by referring them to relevant health personnel and the child welfare system in the community. Despite this opportunity, little is known about how GPs can contribute to this important aspect of preventive care. This study explores GPs’ thoughts and experiences with handling the special needs of these children in general practice.

## Material and methods

A qualitative approach was chosen because we wanted to explore the participants’ thoughts and experiences. The scope of the question is rather narrow and focused, and therefore focus-group discussions were chosen as the method for collecting data [16]. The potential of the group interview is to create a situation where the informants discuss the topics between them and in that way open up to new knowledge.

### Data collection

*Participants*. We wanted to elicit the experiences of GPs with a certain amount of exposure to the research topic. One inclusion criterion was that the GP should have had at least five years in general practice. We invited established continuing medical education groups of GPs in western Norway by mail to the interviews. The four invited groups, a total of 27 GPs (Table I), all agreed to participate. We wanted a strategic sample [17] of informants with respect to gender and rural vs. urban practices.

### Method

*Focus groups*. Group members were encouraged to bring their own stories of clinical encounters with parents who had mental illness, substance abuse, or severe somatic disease. The discussions were led by the first author (FG) with the last author (MH) as a moderator.

The interviews were conducted from February to June 2011, each lasting approximately 90 minutes. During the fourth interview, few new themes were brought up, and we concluded that we had sufficient material for satisfactory analysis.

**Table I.** Overview of the informants.

	Group 1 (Urban)	Group 2 (Rural)	Group 3 (Urban)	Group 4 (Rural)	Total
Number of informants	5	5	9	8	27
Men/women	5/0	4/1	9/0	0/8	18/9
Age (years) Mean Range	56 53–60	56 51–63	58 52–65	46 38–55	54 38–65
Time in general practice (years) Mean Range	21 11–33	26 24–28	26 15–30	14 6–25	22 6–33

The main topics for the focus groups included the following:

1. The sharing of thoughts related to actual cases the GPs had been involved in.
2. Experiences regarding talking to parents in the target groups about how their illness might affect their children.
3. Experiences regarding talking to children about their parents’ health problems.
4. With whom did they collaborate regarding children at risk?

The informants knew each other well and engaged in the discussions of the stories and the questions with reflections, associations, and opinions.

### Analysis

The four focus-group interviews were audio- recorded and transcribed by FG. To manage the data, NVivo9 computer software was used. The material included reflections and case stories. There were seven stories concerning parental problems of abuse, 15 about somatic disease, and 23 about psychiatric disease.

We used thematic analysis [18] to elaborate our results through the following steps:

1. We read through the whole material, obtaining an overview.
2. We identified aspects in the data relevant to the study and coded for relevant patterns or themes.
3. The codes from phase 2 were sorted and condensed into more overarching themes.
4. These themes were reviewed, refined, and validated in relation to the whole data set.
5. Lastly, we defined and decided on the final themes.

The analysis was done in ongoing discussions with the members of our research group, other researcher networks, and GPs with field competence by presenting preliminary results in meetings and courses. This was done to find alternative interpretations of the data and validate our results so far [19,20].

## Results

Two major themes emerged through our analysis:

- Opportunities: what GPs said they could do to help children as next of kin.
- Barriers: what the GPs said were limitations in helping children as next of kin.

### Opportunities

*Identifying children at risk*. Some of the GPs, especially those working in small communities, described how well they knew the family, how they might be familiar with the extended family and social networks, and how they used this information to evaluate whether or not the children were at risk:

“As a doctor in rural districts, you know how many kids your patients have. You know how old they are, you know who their friends are and you know their grandparents. You also know which sports teams they belong to. You get a good overview of the entire family when you've worked a few years.”

Other GPs pointed out how their knowledge about the child was restricted to what the adult told them in a consultation. Many significant things might happen in a child's everyday life that the GP does not know, including troublesome conditions in the child’s environment:

“You may not know how people are when they're not in your office. There they are in a fairly solemn location, and they behave nicely like they've learned. We can't see deviant behaviour, that is easy to hide from a GP.”

The informants said that it was easier to become aware of the children during house calls. Home visits were most often performed when the problem was severe somatic disease or sudden death. They also stated that the threshold for remembering the children's condition often was lower when they worked in preventive health services for children and adolescents.

The GP could be in a good position to identify children at risk, but awareness of the children largely depended on the conditions the GP worked under. Awareness seemed to be easier for those working in small communities, who do house calls, and who work together with health visitors.

*Supporting the patients in the parenting role*. The informants stated that generally they informed and advised either the parent with problems and/or the healthy parent on how to talk to their children and help them. They spoke to a lesser extent with the children themselves and some wanted more education and tools to make conversations with children easier. Some described following up on teenagers with an individual talk. Several informants actively called on families during acute crises, such as sudden death, and could then also speak with the children. One GP spoke of her role in the case of a drug-abusive father's death due to an overdose:

“The mother had asked me for advice on how to inform her son about the father's drug problem. She had told her son that this was an illness and a drug abuse problem, and then the father died of an overdose. Afterwards I gave advice on how to tell the son about the death and why it happened. Naturally, I also visited their home a few times after he died and talked with the little 7–8-year-old boy. It was not easy!”

Many of our informants expressed confidence in relation to informing and advising parents, but they were more uncertain about talking directly to the children.

*Multidisciplinary collaboration*. Most of the GPs recounted how they took part in multidisciplinary meetings in addition to much informal local cooperation regarding patients. In meetings concerning children the doctors said they sometimes had an important role as the parents’ supporter. Several spoke about well-functioning division of tasks, especially with health visitors and GP colleagues. From the same example above, death due to overdose:

“In this case, too, we had good support from the school nurse. After the father died, she came to the school and informed the class, and afterwards she followed up the son and some of his friends. Hence, the role of the school nurse is vital.”

By collaborating with other health professionals, the GPs could help children as next of kin. Some of the informants, however, said that they were seldom involved in inter-disciplinary cooperation, and some spoke about negative experiences, in particular with the child welfare services.

### Barriers

*Lack of time and capacity – barriers within the framework of the consultation*. Most of the informants worked in busy general practices with brief consultations, and they saw most of their patients in the doctor's office. Many of the GPs described it as difficult to bring in the children's situation and their legal rights within the framework of an ordinary consultation. It was usually the patient who introduced the subject of the consultation, and the possibility for the GPs to bring in other topics was perceived as limited. The informants spoke about time and capacity limits, as illustrated here:

“In that family there were children who were affected by the father's alcohol abuse, but how I could help the children I could not imagine. I spent plenty of hours with that man, long consultations every week about his problems. This theme, to see and help the patient's children, gets lost in all other things we have to do.”

Some also stated that they purposely omitted addressing the children's situation because they were afraid of being left with too much work on their hands that they did not know how to handle. The doctor's feeling of responsibility for the actual children was often unclear because the family members might be on the lists of various GPs.

FG: “Does the general practitioner already have so many tasks that this becomes difficult to handle during the workday?”

GP: “I think that's a good point, especially in a situation where the rest of the family is not on your list. Then you think there are other people involved who will take care of them. This can be people you don't know and whom you have never seen.”

In some of the stories the GPs mentioned informal contact between colleagues concerning the target families, but in many cases it was evident that no GP actually took responsibility for the child at risk.

The frameworks within general practice in terms of consultations and the list system can leave the children invisible when parents are in contact with their doctor.

*Doctors are afraid of hurting and losing their patients*. Some GPs said they avoided addressing the children's situation in consultations with ill parents. They were afraid the patient would leave their list and choose another GP, and that this would be a disadvantage for both the patient and his or her family. Some were also afraid that introducing this theme would increase feelings of guilt and make the burden greater on parents who were already struggling. One doctor expressed it thus, when asked about how to thematize the children's situation:

“It is difficult, because then it's as though I am also saying that her problem is her children's problem. Then I am putting the blame on her, and here she has come to get help for herself. I am just placing one more burden on her shoulders, I should think.”

The doctors appeared to be confident in their supportive role in cases of acute illness. They said that helping a family in a crisis, children and adults alike, was a natural part of the tasks a patient would expect their GP to undertake. The GPs seemed more uncertain that parents with mental health and substance abuse problems accepted that giving support to their children was a natural task for their GP.

## Discussion

The aim of our study was to investigate how Norwegian GPs support children as next of kin and their families. The results showed that they might be in an important position to support the children, but often missed the opportunity to act.

The working conditions in Norwegian general practice can represent barriers to the support of these children. Three issues *–* the invisible children, the trustful but limited relationship, and the under-utilized consultation – form the basis of the following discussion.

### The invisible child

Within the framework of the consultation it seems to be difficult for the GP to become aware of and help the child of a patient with problems. If the GP does not know the family situation, the doctor gets to know only what the patient tells her or him. In Norway, the children are usually not registered in their parents’ dossier. It is easy for the patient to hide or forget to bring up information about their children. And if the GP does not ask, important information about the child can be missed. This is especially apparent when the child has a different GP than the parent. It seems easier to address the issue when the doctor leaves the office for home calls or works in collaboration with others in the municipal health service, such as the health visitor. The same findings were found in a study of GP services for children and young people with mental health difficulties [21].

### A doctor–patient relationship with limitations

Several previous studies have shown that trust is a key factor in the doctor–patient relationship [22,23], and patients often have great confidence in their GP. Patients have reported that a trusting relationship can make it easier to bring up sensitive themes [24]. This has also been found with battered women, who have to be convinced of loyalty from their physicians before they admit to having been abused [25]. In our material we had reports from experienced doctors, some of whom had practised in the same community for a long time. These GPs had a long-term relationship with their patients and had prime conditions for building trusting doctor–patient relationships. Nevertheless they told stories of where they did not rely on the alliance. Some GPs were afraid of giving the patient even more burdens by increasing feelings of guilt and shame. Helping children as next of kin might introduce a sensitive theme for the patient. In general practice the doctors often have to address sensitive topics, such as concerns about the patients’ weight or alcohol abuse [26]. Even if the GPs consider lifestyle changes important, several studies report that follow-up is deficient. Doctors often explain that they will not jeopardize the relationship by bringing up such a sensitive topic, which is similar to our findings [27–29]. Fear of interrupting the therapeutic alliance is also found to be a barrier to psychiatric work being family focused [11].

### The under-utilized consultation

In the patient-centred consultation model, the physician strives to interpret the patient's illness and problems within his or her own frame of reference, and the patient plays an active role in the consultation [13]. According to McWhinney, frames of reference for the patient also include the family situation. This implies the doctor needs to thematize the family, including the children. When avoiding introducing the children's situation at all, the GP does not utilize the possibilities given by the consultation model. However, the patient-centred method is not always sufficient for the GP to do his or her job, since the method implies that the child is not included as part of the consultation unless the patient follows up the issue. Even if the parent does not welcome a conversation about the child's needs, the GP has an ethical and legislative requirement [9] to address his or her concerns and ask for the parent's consent to ensure that the child get adequate information and follow-up.

### Methodological discussion

We established a strategic sample with spread based on the selection criteria, but all groups turned out to be skewed concerning gender. We do not think this influenced the discussions in ways that distorted the results significantly.

The participants were recruited from established groups, and they knew each other well. It might be that the doctors wanted to tell more success stories and speak less about the times they did not contribute or failed [30]. This trend might have been strengthened by the fact that FG is a GP and MH a child and adolescent psychiatrist [31]. However, since our primary goal was to find out how GPs may contribute, we believe the results are valid for this purpose [16]. We wanted a sample of experienced GPs, and age and time in practice is therefore higher than average among GPs. Our results therefore cannot be generalized to young, more inexperienced GPs without reservations.

## Conclusion

Norwegian GPs may be in a good position to support children as next of kin, but they also face barriers in doing so. The children easily remain invisible in the GP's consultation with their parents. Time constraints as well as fear of jeopardizing the patient–doctor relationship may cause the GP to hesitate in bringing up this sensitive theme. From the experience in this study it seems as if support from the GP to a child who is burdened as next of kin depends often not on careful consideration of what is best for the patient and the child in the long run, but more on short-term convenience reasons.

## Implications and further research

It is an important preventive mental health task for the GP to identify, inform, and ensure follow-up for children as next of kin. The present study introduces hypotheses that might be useful in the development of tools and guidelines for GPs to perform these tasks. To further enquire about the possible role of GPs, information is also needed from the perspective of the parents and the children.

## Ethical approval

According to the Regional Committee for Medical and Health Research Ethics, Western Norway, the Act does not apply to this project.
